# Identifying local structural states in atomic imaging by computer vision

**DOI:** 10.1186/s40679-016-0028-8

**Published:** 2016-11-02

**Authors:** Nouamane Laanait, Maxim Ziatdinov, Qian He, Albina Borisevich

**Affiliations:** 1 Institute for Functional Imaging of Materials, Oak Ridge, 37831 TN USA; 2 Center for Nanophase Materials Sciences, Oak Ridge, 37831 TN USA; 3 Materials Sciences and Technology Division, Oak Ridge National Laboratory, Oak Ridge, 37831 TN USA

**Keywords:** Scanning transmission electron microscopy, Scanning tunneling microscopy, Computer vision, Unsupervised machine learning

## Abstract

The availability of atomically resolved imaging modalities enables an unprecedented view into the local structural states of materials, which manifest themselves by deviations from the fundamental assumptions of periodicity and symmetry. Consequently, approaches that aim to extract these local structural states from atomic imaging data with minimal assumptions regarding the average crystallographic configuration of a material are indispensable to advances in structural and chemical investigations of materials. Here, we present an approach to identify and classify local structural states that is rooted in computer vision. This approach introduces a definition of a structural state that is composed of both local and nonlocal information extracted from atomically resolved images, and is wholly untethered from the familiar concepts of symmetry and periodicity. Instead, this approach relies on computer vision techniques such as feature detection, and concepts such as scale invariance. We present the fundamental aspects of local structural state extraction and classification by application to simulated scanning transmission electron microscopy images, and analyze the robustness of this approach in the presence of common instrumental factors such as noise, limited spatial resolution, and weak contrast. Finally, we apply this computer vision-based approach for the unsupervised detection and classification of local structural states in an experimental electron micrograph of a complex oxides interface, and a scanning tunneling micrograph of a defect-engineered multilayer graphene surface.

**Electronic supplementary material:**

The online version of this article (doi:10.1186/s40679-016-0028-8) contains supplementary material, which is available to authorized users.

## Background

A multitude of imaging probes such as scanning transmission electron microscopy (STEM) have reached the requisite spatial resolution, at least in two dimensions, to directly distinguish the individual structural microstate of a material, namely an atom and its local neighbors [1]. In addition, the prevalence of auxiliary information channels such as electron energy-loss spectra acquired at similar spatial resolutions allows one to append to these structural microstates additional chemical/electronic state information [2–4]. The data that emanate from such modalities reveal a wealth of information regarding the static modulation of material properties by local structural deviations [5], competing structural ground states [6], and even dynamic phase transformations or ensuing structural reordering during in situ atomic resolution imaging of materials growth [7]. These imaging modalities are crucial to fundamental investigations of modern materials, which often display a range of structural configurations and order parameter phases. In many cases, some structural phases are not directly discernible by the diffraction-based methods of X-rays and neutron scattering [8, 9] due to either their small volume fraction and/or their lack of long-range periodicity, and therefore require an imaging approach [10, 11] for identification.

To identify and classify local structural states and their correlations as resolved by atomic resolution imaging, the traditional language of crystallography with its restrictive assumptions of symmetry and periodicity leaves much to be desired [12]. Nevertheless, many successful approaches that extract structural information from atomically resolved data [13, 14] still adopt many of the underlying assumptions of traditional crystallography, through the use of integral transforms such as Fourier transforms (e.g., in geometric phase analysis [15]) and other techniques from harmonic analysis. Such techniques explicitly transform the local spatial information into a space that presupposes the presence of a coherent superposition of components to classify the structural states present in an image. Recent work has taken a different route to identify local structural states by analyzing the intrinsic intensity signatures in atomically resolved images through multivariate statistics [16]. The feature identification method used is strictly local, however, and does not incorporate the information present in neighboring intensity distributions around an atom or defect site. Here, we explore an alternative method to identify and classify local structural states in atomically resolved images that is rooted in a multi-scale extraction and classification of structural states present in an image. The presented approach, in essence, provides a middle ground between structure identification that relies on “single-point” intensities and those that analyze information obtained from an extended region through integral transforms.

The underlying assumptions of the presented approach are contextual information and scale invariance. The former implies that the local intensity distribution in the neighborhood of a particular structural state, e.g., atomic coordination surrounding a defect site, is the key measure by which we perform detection of local structural states. Furthermore, to not assume a priori, the spatial extent of these local states our approach should be scale invariant, whereby we would like to detect not only atoms but also clusters of atoms whose intensity distribution becomes more localized at larger length scales in the image (i.e., obtained through progressive down-sampling).

Our methodology borrows heavily from techniques developed in the field of computer vision to perform tasks such as pattern recognition, through the use of a scale-invariant feature detectors and descriptors [17]. Following detection, we classify the structural states by a hierarchical clustering strategy [18, 19] using the scale-invariant descriptor associated with each state. We tested the fundamental assumptions of our approach, namely scale invariance and contextual information, by applying it to simulated scanning transmission electron microscopy images of ideal crystals and atomically sharp interfaces between crystals. To explore the utility of this analysis in practice, we performed an extensive quantitative study of the accuracy in detection of local structural states in the presence of instrumental factors such as noise- and material-dependent factors such as low contrast, finding that this approach is robust under common experimental conditions. Finally, we conclude by demonstrating automated extraction and classification of local structural states in STEM images of strained interfaces of SrTiO_3_/LaCoO_3_ and local modulations in the electron density of states near defects on graphite surfaces imaged by scanning tunneling microscopy.

## Methods

In what follows, we restrict our attention to 2-dimensional atomically resolved images with gray scale value, where the image *I* is defined as a mapping from a 2-dimensional spatial domain ***x*** (i.e., pixels) to a strictly positive real number (i.e., intensity): $I{:}\,{x} \to {\mathbb{R}}^{ + }$. Feature detection proceeds by locating keypoint features, denoted by *Kp(* ***x***), in an image *I*(***x***), that are extrema of a detector function *F*(*ζ*, ***x***), where *ζ* is a parameter or set of parameters that specify the feature detector. The detector function is an operator that transforms the image locally, and often involves spatial derivatives of the image. A keypoint can be then generally expressed as

1 $$\begin{array}{*{20}c} {Kp = \text{argmax}_{{\zeta ,{\text{x }}}} \left( {F \circ I} \right)\left( \varvec{x} \right)\;\;\text{or} \;\; \text{argmin}_{{\zeta ,{\text{x }}}} \left( {F \circ I} \right)\left( \varvec{x} \right) } \\ \end{array}.$$

Numerous feature detection methods have been developed in the field of computer vision that achieve scale invariance [20, 21]. Here, we restricted our attention to the Laplacian of Gaussian operator (LoG). The latter is one of the most widely used feature detectors and defined as

2 $$\begin{array}{*{20}c} {F\left( {\sigma ,\varvec{x}} \right) = \nabla_{\text{x}}^{ 2} G\left( {\varvec{x},\sigma } \right)} \\ \end{array},$$

where *G*(.) is a multivariate Gaussian distribution with variance $\sigma$ and ∇^2^ is the Laplacian operator, evaluated in the spatial domain of the image.

The LoG operator is efficient in detecting local intensity curvatures in images. Given that atomically resolved images show pronounced local intensity curvatures, we use the LoG throughout as a detector to extract features. As first pointed out by Lindeberg [22, 23], the Laplacian of Gaussian kernel provides a natural way to extract keypoint features that are stable in both the image spatial domain and the scale space of the image. The latter is constructed by consecutive blurring (convolution with a Gaussian filter) and down-sampling of the original image I(*x*) [23]. With additional approximations in regard to the detector function, the construction of a scale space, and search strategies for the extrema in the spatial and scale domains, Lowe constructed a feature extraction and description framework known as the scale-invariant feature transform (SIFT) [24]. SIFT is widely regarded as one of the most effective detector-based feature extraction techniques with wide-range applications from pattern recognition [25] to image registration [20], and was used throughout this work as descriptor for a local structural state.

## Results

### Scale-invariant detection and description of structural states

We used simulated electron microscopy images of bulk SrTiO_3_ and SrTiO_3_/BaTiO_3_ interface projected on the [100] direction. The images were generated using an implementation of the standard multislice code using standard imaging conditions for Nion UltraSTEM200 for 200 kV operation and an aberration-free probe [26].

**Fig. 1:**
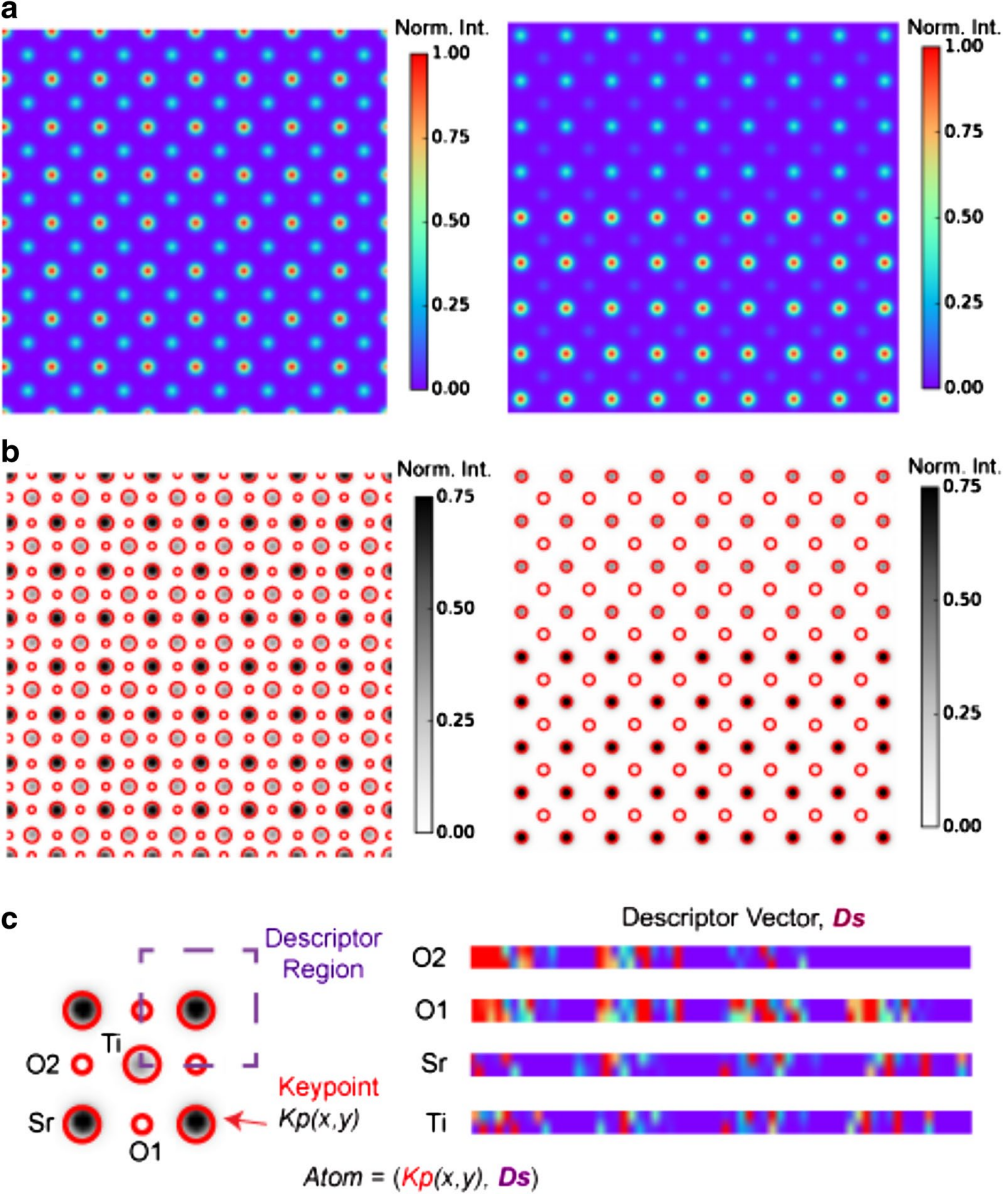
Structural states as scale-invariant features. **a** Simulated STEM images of bulk SrTiO_3_ and SrTiO_3_/BaTiO_3_ interface with the electron beam propagating along the [100] crystallographic direction. Images are convoluted with a Gaussian function with FWHM of 0.7 Å to account for the finite source size of the electron beam. **b** Features extracted by the Laplacian of Gaussian detector are shown as an overlay of *circles* on the images in **a**. The intensity scale was inverted to improve the visibility. The size of the *circle* indicates the scale at which the feature was detected. For simplicity in the ensuing analyses, the contrast threshold of the LoG is tuned so that oxygen columns in the right image in **b** are not detected (see Additional file 1 for all atomic columns). **c** Close-up of the *left image* in **b** indicating both the keypoint, *Kp,* which describes the atom locally and the descriptor vectors, ***Ds***, which encode the intensity distribution of neighboring columns to provide a nonlocal description of the column. Descriptors for the different atomic columns are shown as 1-dimensional vectors, indicating that columns with the same intensity can have different descriptors due to the different angular configuration of their neighboring atoms. The structural state, in this case an atomic column, is then defined by the pair composed of (*Kp*, ***Ds***). The implementations of the LoG detector in the Python scikit-image library [41] and SIFT in OpenCV [42] were used throughout

The raw simulated images were convoluted with a Gaussian probe size with a full-width-half-max of 0.7 Å to account for the finite source size of the electron beam. No other preprocessing of the images was performed. A global scaling of the intensity was applied. This intensity scaling has no effect on the Laplacian of Gaussian detector, since the detector is only sensitive to the local image contrast gradient (Fig. 1a).

The atoms detected in each image by the LoG are indicated by circles. The size of each circle is proportional to the scale (i.e., *σ*) at which the feature was found to be an extrema of the LoG operator (Fig. 1b). Note that while the oxygen columns in the bulk SrTiO_3_ are not clearly evident in Fig. 1a due to their low intensity relative to Sr and Ti, they are readily detected by LoG albeit at a smaller scale than either Sr or Ti columns. The detected feature is commonly referred as a keypoint, *Kp,* in computer vision. Associated with each keypoint are the coordinates of the feature (*x,y*) and its scale (Fig. 1c), as well as other properties that we do not make use of in this work.

Given a particular *Kp*, we use the scale-invariant feature transform to compute a descriptor, ***Ds***. The descriptor is centered around *Kp*(*x,y*) and encodes the intensity distributions around that feature (Fig. 1c). Both the spatial extent of ***Ds*** and the intensities it contains are sampled from the spatial domain of the image but at the appropriate scale. Consequently, the image patch from which ***Ds*** is extracted (16 × 16 pixels centered on *Kp*(*x,y*)) varies in size with respect to the spatial domain in the original image. The SIFT descriptor is composed of intensity gradient magnitudes and orientations that are appropriately weighted to decrease their contribution to the descriptor as a function of their distance from *Kp*(*x,y*) [24]. Furthermore, the intensity values in ***Ds*** are transformed to a local frame of reference, i.e., with respect to *Kp*(*x,y*) The latter provides a description of the feature that is rotation invariant and reduced sensitivity to global changes in imaging conditions such as illumination [17, 27]. The resultant SIFT descriptor is a 128-dimensional unit vector and is shown in Fig. 1c in a vector format for the different detected columns in Fig. 1b. In this work, we modified the SIFT descriptor, by intentionally breaking its rotational invariance through a choice of a preferred orientation angle of the ***Ds*** image patch (0° defined with respect to the *x*-axis of the image) (see Fig. 1c). This modification leads to a minimalistic descriptor that is only translation invariant and does not incorporate other symmetry assumptions. Consequently, ***Ds*** provides a distinct description of intensity gradients that are dissimilar for atomic columns such as O1 and O2 despite them having identical local intensities, since their neighboring columns (Sr, Ti) are in a different orientation order. Given *Kp* and ***Ds***, we then define a structural state,

3 $$\begin{array}{*{20}c} {S = \left( {Kp,\varvec{Ds}}\right)}, \\ \end{array}$$

as a pair composed of a keypoint, which gives a local description of the image intensity, and ***Ds*** which provides a *nonlocal* description of neighboring intensity gradients. This description of a structural state, such as an atomic column, is both scale invariant and context dependent.

### Noise and contrast behavior of structural state detection

**Fig. 2:**
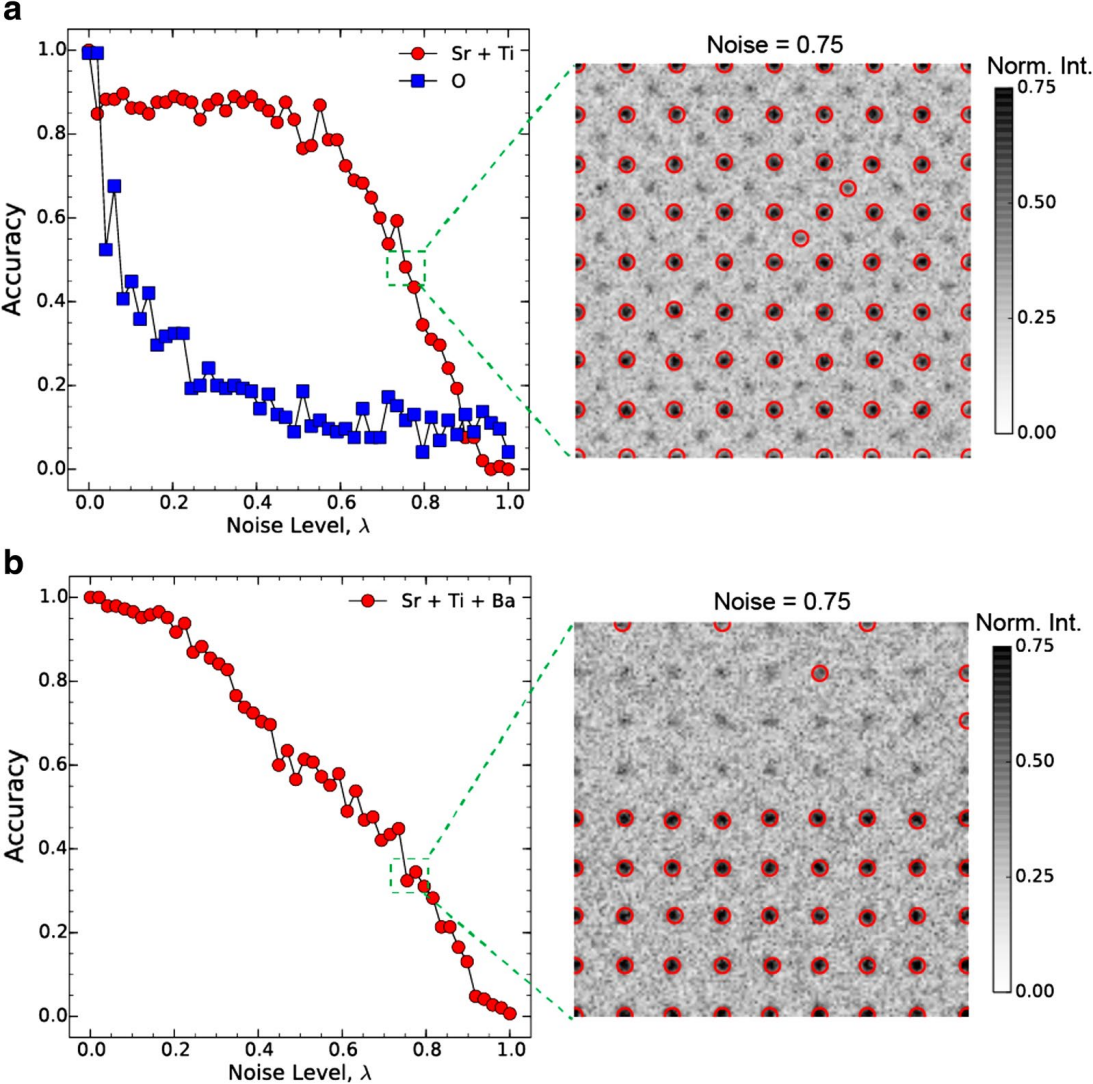
Atomic column detection in the presence of noise and low contrast. The accuracy of atom detection is analyzed as a function of noise level, *λ*. The noise, sampled from a Poisson distribution, is added to the STEM simulated images of bulk SrTiO_3_ **a** and SrTiO_3_/BaTiO_3_ (**b**). The accuracy is computed by comparison of detected features *Kp*(*x,y*) at some $\lambda \ne 0$ to the ideal images (*λ* = 0). To demonstrate the dependency of atom detection on the contrast of the atomic column, the accuracy of the detection of O columns and “Sr + Ti” is calculated and shown separately in (**a**). The images indicate the detected atomic columns at the corresponding noise levels. For ease of comparison with experimental images, the noise level shown here is defined as a fraction of the highest scattered intensity (from an atomic column) that is present in the simulated image. Due to the projective nature of STEM imaging, Ti columns are in fact not pure Ti columns but mixed Ti and O columns

We assumed that the imaging is free from all geometric distortions due to scanning of the electron probe, and focused on testing the robustness of the above formulation at different noise levels and local contrast values. Each simulated STEM image (Fig. 1a) is altered with noise that is sampled from a Poisson distribution and added in a linear convex fashion to the ideal image, with the noise level given by *λ*. The accuracy of the atomic column detection as a function of *λ* is calculated by direct comparison to the ideal case (i.e., *λ* = 0, accuracy = 1). Furthermore, in the case of bulk SrTiO_3_, we split the accuracy into two classes depending on the local contrast of the detect atoms. We found that a detection accuracy of Sr and Ti atoms fluctuates about 0.85 (±0.06) for *λ* ≤ 0.4, and falls off precipitously for *λ* > 0.4. As expected, local intensity fluctuations affect the detection of Ti atoms first, as shown in Fig. 2a. The detection accuracy of O atoms, on the other hand, becomes unreliable for noise levels that even exceed 0.05 due to their low contrast values (<0.05). Such behavior is well known in experimental *Z*-contrast STEM images [28], where oxygen columns, while in principle resolvable, are often not detectable due their weak Rutherford cross-sections relative to heavier atoms and the finite dynamic range of the detector. The detection accuracy of Sr, Ti, and Ba columns in the simulated image of SrTiO_3_/BaTiO_3_ as a function of noise level behaves in an analogous manner to simulated bulk SrTiO_3_. Robust image de-noising strategies can, of course, be employed in practice to increase the accuracy of atomic column detection by the LoG detector, but this was not performed here as the de-noising constitutes a separate problem from the focus of this paper, and is well covered in both electron microscopy and image recognition literature.

The primary reason for the reduced accuracy in detected atomic columns is the delocalization of their response to the LoG kernel in scale space [29]. Note, however, that the LoG detector has a strong response to features near edges (of an image), which, in practice, can lead to an overestimation of the detection accuracy. From the above analysis, we conclude that for noise levels $\lambda < 0.4 C_{\rm{max}}$, where *C*_max_ is the maximum image contrast of the structural feature of interest, the presented approach can produce a meaningful and robust detection. An additional aspect of the LoG worth mentioning is that the presence of other instrumental factors, such as blurring, only affects the scale at which the feature is detected, but not the accuracy of the LoG detector. Finally, we emphasize that the LoG searches for both maxima and minima in the local imaging contrast as a function of scale and therefore can be used to detect missing atoms or used in imaging modes such as bright-field imaging where atomic columns can also be represented by the image minima. In such an instance, its detection robustness will be affected by the presence of noise in a manner similar to the above analysis.

### Structural state classification

The definition of a structural state in Eq. 3 allows us to classify the different detected atomic columns to find the main structural classes present in a particular image. Numerous methods exist to perform these classification tasks. Here, we focus on unsupervised machine learning to explore the effectiveness of the presented approach to “learn” the overall structural configuration in a material. To that effect, we use hierarchical agglomerative clustering.

In agglomerative clustering, each structural state *S* is initially considered to belong to a distinct class ${\mathbf{\mathcal{C}}}_{\text{i}}$. Following this initial assignment, different classes ${\mathbf{\mathcal{C}}}_{\text{i}}$ and ${\mathbf{\mathcal{C}}}_{\text{j}}$ are merged into a new class ${\mathbf{\mathcal{C}}}_{\text{k}}$ if their respective members (i.e., structural states) are similar, given some notion of similarity, *g.* In our case, the similarity (or affinity) measure between two structural states, *S*_*i*_ and *S*_*j*_, is naturally defined by the (Euclidean) distance between their respective descriptors, ***Ds***_i_ and ***Ds***_*j*_,

4 $$\begin{array}{*{20}c} {g\left( {\varvec{S}_{i} ,\varvec{S}_{j} } \right) = \varvec{Ds}_{i} - \varvec{Ds}_{j}^{2},} \\ \end{array}$$

and is used to merge the different structural classes. Different methods, known as linkage, apply the similarity measure to the classes in a specific way. We use the average linkage method which uses the average similarity between classes:

5 $$\begin{array}{*{20}c} {\bar{g}\left( {{\mathbf{\mathcal{C}}},{\mathbf{\mathcal{D}}}} \right) = \frac{1}{{N_{\text{C}} N_{\text{D}} }} \mathop \sum \limits_{{i \in {\mathbf{\mathcal{C}}}}} \mathop \sum \limits_{{j \in {\mathbf{\mathcal{D}}}}} g\left( {i,j} \right),} \\ \end{array}$$

where *N*_C_ (*N*_D_) are the number of structural states belonging to each class ${\mathcal{C}}\left( {\mathcal{D}} \right)$. With $\bar{g}$ as similarity measure, agglomerative clustering results in a classification that groups structural states into relatively compact classes that are well separated [30]. The only remaining parameter that must be specified to perform the hierarchical clustering of structural states is the level at which we must truncate the merging procedure. This was determined by a statistical measure that optimizes the similarity between structural states that belong to the same class (see Additional file 1 for additional details and illustration of this analysis for the classification used here, Additional file 1: Fig. S2).

**Fig. 3:**
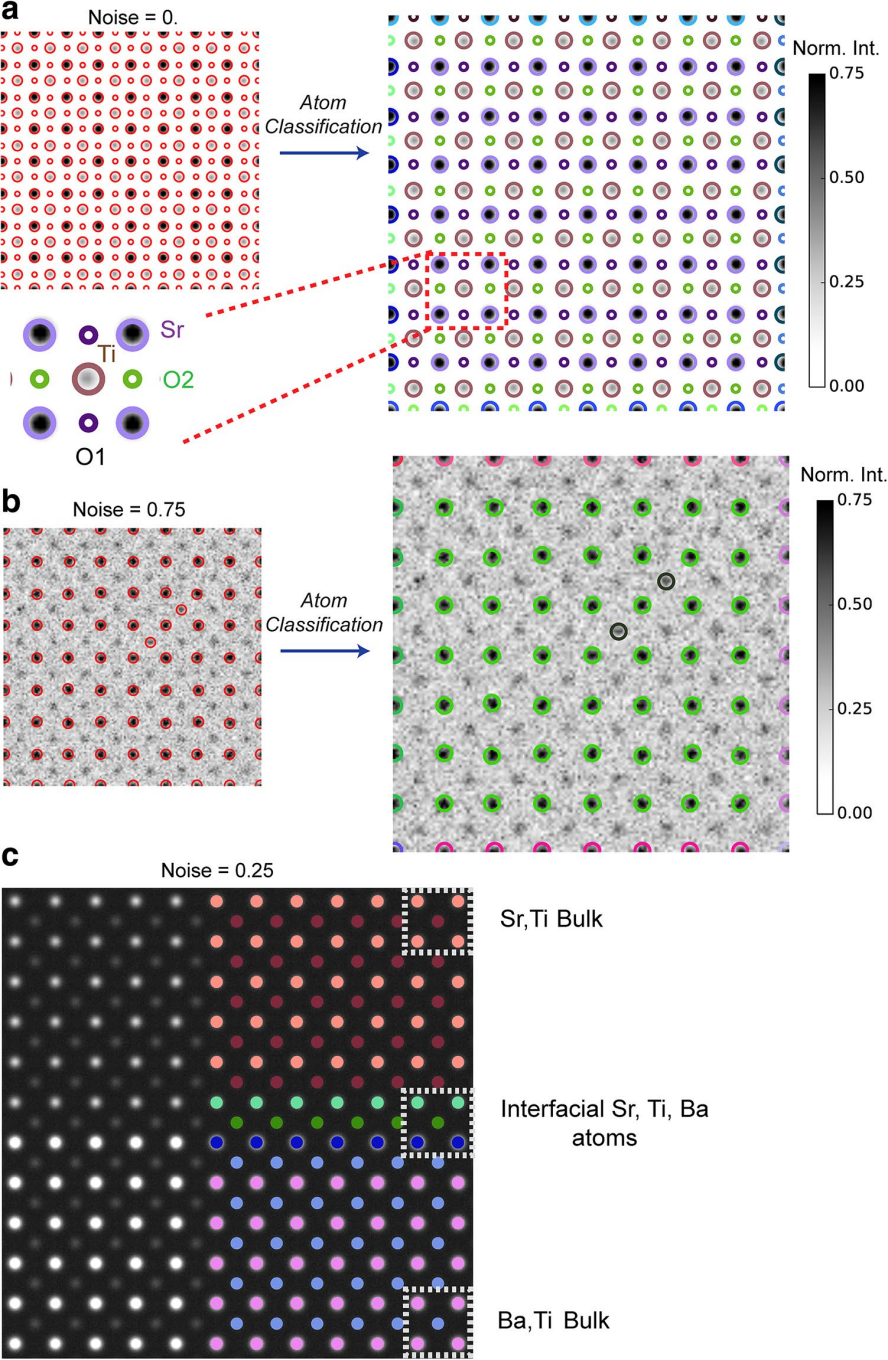
Classification of Local Structural States. Detected atomic columns at different noise levels in simulated STEM images are classified by hierarchical clustering, with different structural classes represented by circles with different colors. The different atomic columns in the [100] projection of the SrTiO_3_ unit cell are all classified as distinct structural states by the presented approach (**a**). In the presence of noise, the distinction between Sr and Ti atomic columns is still maintained (**b**). Note that Sr atoms at the edge of the image belong to separate classes since their coordination is different than that of Sr atoms in the “bulk”. **c** Classification of atoms in the image of a SrTiO_3_/BaTiO_3_ interface distinguishes the interfacial atoms (Sr, Ti, Ba) from those present in the bulk phases, and provides a complete description of the structural configurations present in the image

The results of the classification of atomic columns in the SrTiO_3_ and SrTiO_3_/BaTiO_3_ images (shown in Fig. 1b) using agglomerative clustering at various noise levels are shown in Fig. 3, where each structural class is represented by a different color coding. For bulk SrTiO_3_, we find that the classification clearly distinguishes between the different atomic columns in the unit cell. Note that although O1 and O2 oxygen columns have identical imaging intensities and are equivalent under the rotational symmetry of SrTiO_3_ (*P2* mm), they are grouped into different clusters, since their descriptors are not rotationally invariant as discussed above.

We found that even in the presence of large noise levels (*λ* = 0.75), columns of different types (Sr, Ti) are still classified separately, giving good evidence of the robustness of Eq. 3 in the presence of noise. In the case of SrTiO_3_/BaTiO_3_, a complete classification of the unit cell configurations is achieved, with Ti columns in bulk STO, at the interface, and in bulk BTO grouped as distinct states. Similar results are also obtained for Sr and Ba atomic columns. These observations are crucial evidence that the definition of an atomic column given in Eq. 3 does encapsulate the local coordination environment necessary to discriminate between different structural states and further reinforce the utility of formulating a structural state as a combination of local and nonlocal image intensities.

### Strained interfaces and defects

We illustrate the utility of the structural state extraction and classification in experimental images by two case studies from some of the most widely used atomic imaging modalities, namely scanning transmission electron microscopy data of interfaces in heteroepitaxial systems and scanning tunneling microscopy (STM) data of defect states found on the surface of graphite.

**Fig. 4:**
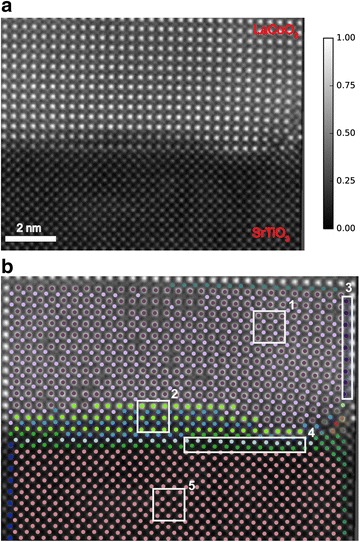
**a** HAADF-STEM images of LaCoO_3_/SrTiO_3_ interface. The color scale in normalized intensity. **b** Classified structural states clearly highlight the diffuse nature of the interface, with each boxed region outlining a particular structural configuration: *1* bulk LaCoO_3_, *2* Interfacial LaCoO_3_, *3* distorted column of Co atoms, *4* interfacial SrTiO_3_, *5* bulk SrTiO_3_

In studies of solid/solid interfaces, in particular those interfaces that originate through epitaxial growth, characterizing the structural nature of the interface is crucial to tailoring the materials properties. For instance, solid/solid interfaces are often the starting point of extended defects such as misfit dislocations that arise to compensate epitaxial strain, and lead to elastic fields propagating in both directions from the interface, substantially modifying its crystal structure and potentially its properties. It has also been demonstrated that, even for a case of coherent epitaxy, the different symmetry of the film and substrate can result in a progression of distinct structural states localized in the vicinity of the interface [31]. In all these instances, it is crucial to precisely extract and identify the local structural states present at interfaces. We applied the presented approach to a *Z*-contrast STEM image of SrTiO_3_(STO)/LaCoO_3_ (LCO) interface. This image was acquired using Nion UltraSTEM 100 operated at 100 kV (Fig. 4a).

The classification of structural states leads to a succinct representation of the evolution of structural states at the interface, as represented by classes of LaCoO_3_ unit cells (region 1 in Fig. 4b) that are clearly distinct from their bulk phase (region 2). By capturing these structural deviations in LCO that span multiple unit cells, our approach produces an automated and unsupervised technique to determine the extent of the interfacial structural states (compare Figs. 4b to 3c). Large structural distortions in a column of Co atoms (region 3) are also singled out by the classification as a distinct structural state and represent the elastic effects that originate at a defect at this incoherent interface and propagate far into the bulk phase. For STO 2, atomic planes were identified as separate structural (region 4) classes than the bulk (region 5). Given this classification of the atomic columns in this system, additional properties (e.g., displacements of Co with respect to the center of LCO unit cell) can be then readily computed for a structural class and compared to others to fully characterize the nature of the interface in this system.

Next, we extract and classify structural states that arise due to point-like defects on a graphite surface. Point defects such as monovacancies, adsorbed atoms, interstitials, and Stone–Wales defects are known to affect strongly the electronic and magnetic properties of graphene layers [32]. Recently, it was realized that the electronic structure of atomic vacancy is highly sensitive to the details of the passivation of its dangling σ bonds with foreign chemical species, such as hydrogen and oxygen [33]. Here, we focus on the so-called V_111_ type of the monovacancy–hydrogen complexes [33, 34]. The V_111_ complex, in which each σ dangling bond is passivated with one hydrogen atom, is characterized by the formation of a localized nonbonding π electronic state at the Fermi level [34] whose decay into the “clean” area of the lattice can be described by *r* ^−2^ law [35]. To date, the studies of monovacancy–hydrogen complexes (as well as other types of point defects) in graphene-like materials have been limited to either the single-layer structure or AB (Bernal)-stacked structure. On the other hand, a rotation of graphene layers with respect to each other, particularly in the case of low twist angles (below 10°), may result in an alternation of the system’s low-energy electronic structure, such as a reduction of the Fermi velocity and associated localization of charge carriers [36, 37], which may in turn alter the electronic and magnetic properties of the vacancy. Below, we analyze the scanning tunneling microscopy (STM) data on hydrogen-passivated single atomic vacancies of the V_111_ type in the topmost graphene layer of graphite that is rotated relative to the underlying layer(s).

**Fig. 5:**
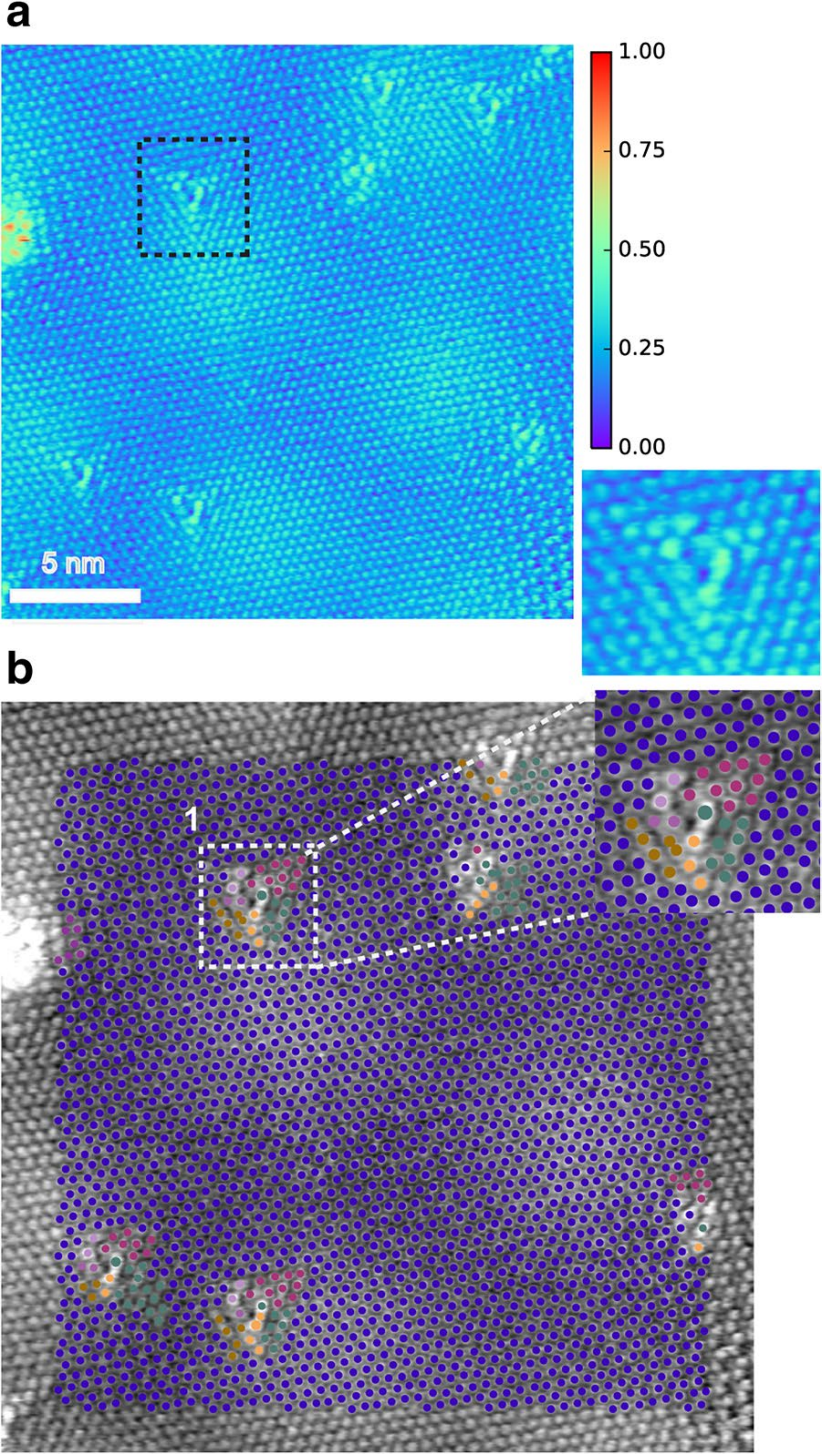
Scanning tunneling microscopy of defects on graphite. The image was acquired with a sample bias voltage of 100 mV and tunneling current setpoint of 0.7 nA. **a** The defects (*box outline*) are monovacancy–hydrogen complexes generated through Ar^+^ ion bombardment, followed by annealing in a hydrogen environment. These defects modulate the local electronic density of states in their immediate vicinity as shown in the inset. The *color bar* is normalized intensity. **b** Extraction and classification of atoms, select edge atoms surrounding a monovacancy–hydrogen complex (e.g., outlined region *1*) as being distinct from the rest of the atoms in the system, with different structural classes encoded by a unique color label

Figure 5a shows the STM image of the topmost graphene layer of graphite that features a well-defined Moiré pattern and is peppered with monovacancy–hydrogen complexes of the V_111_ type. The V_111_ complexes were prepared by sputtering the surface of a graphite sample with low-energy Ar^+^ ions and its subsequent exposure to atomic hydrogen environment and annealing. The choice of experimental parameters was the same as reported in the study of V_111_ complexes in Ref. [34]. The extracted and classified structural states by our methodology are shown in Fig. 5b. First, note that the “edge” atoms around the vacancy produce a strongly nonequivalent response in terms of the corresponding local intensity of the STM signal (see inset to Fig. 5a). Given that the STM signal is a convolution between topographic and electronic features, this in-equivalency may reflect the out-of-the-plane structural distortions at the vacancy site. Our analysis allows the extraction of detailed information on the distribution of the vacancy’s nonbonding state for each V_111_ complex (associated with magenta, green, and orange circles, e.g., region 1 in Fig. 5b). In particular, we found that the distribution of the STM signal associated with the vacancy’s nonbonding state (i) does not follow the threefold symmetry of underlying atomic lattice, which can be related either to the aforementioned structural distortions or to the rotational direction of the topmost graphene layer (nonzero twist angle), and (ii) the details of its propagation appear to be sensitive to the relative position of vacancy with respect to Moiré spots on the surface. To confirm the latter, our analysis must be carried out on a larger set of STM images and sample conditions, and is beyond the scope of this article. Nonetheless, the efficient extraction and classification of structural states associated with the monovacancy–hydrogen complexes represents a crucial first step in a more systematic study of modulating the electronic configurations of graphene through point defects.

## Discussion

A key ingredient to the success of the Computer Vision-based analysis of local structural states resides in the definition of a structural state that combines both local and nonlocal image intensity distributions, in contrast with previous methods that rely on single-point intensities [14, 16]. For instance, a single-point intensity method would not differentiate between the Ti columns present in bulk BaTiO_3_, Ti columns in bulk SrTiO_3_, and those at the interface of STO/BTO, since they all have indistinguishable intensity values, and spatial separations and angles with respect to their neighboring atoms, yet, differ only in the type of atoms that constitutes their coordination (Fig. 3c). The latter is a direct consequence of the definition of a structural state given in Eq. 3, whereby intensity gradients in a neighborhood around *Kp* are encoded in ***Ds***, with the size of this neighborhood directly given by the appropriate scale at which the keypoint was found to be an extremum of the Laplacian of Gaussian detector. Another illustrative example of the advantages of the present approach is in detecting a range of distinct classes in the local configuration of (La, Co) columns at the interface of LCO/STO that clearly reflect the strained nature of the latter. Given the success of our approach in detecting these subtle variations in the structure of materials, it would be interesting to explore in future work if one can reconstruct the fundamental ingredients of the lattice and unit cell directly from the more primitive definition of a structural state in Eq. 3, which relies solely on real space image information and the concept of scale invariance, without relying on the priori knowledge of the average crystallographic symmetry.

The classification procedure used here, namely hierarchical clustering, enabled a physically meaningful categorization of structural states in a number of cases, both for simulated and experimental data. This unsupervised learning approach, however, lacks a clear connection to the physics of the problem. In many contexts, one often seeks the identification/classification of local structural states subject to well-defined physical principles such as spatial connectivity, or localization due to the presence of interfaces, defects, etc. Under these conditions, one can supplement hierarchical clustering with connectivity constraints to generate structural classes that obey a set of physical assumptions. In essence, it allows one to test different physical hypotheses regarding the local structure present in the system at hand.

We have shown that representing a structural state with computer vision-based descriptors that are efficient at encoding image information leads to an analysis approach that can discriminate between the myriad of local states in the presented data across vastly different imaging modalities. The preponderance of atomically resolved images both in the literature and open databases provides an opportunity to begin data exploration of local structural states that are shared by a variety of materials and their evolution during varying experimental conditions. The SIFT descriptor with its scale invariance could provide one of the promising methods by which one can fingerprint local structural states of interest to perform structural recognition against the above databases. Furthermore, the structural identification we presented could also be used to identify recurring artifacts in atomically resolved imaging such as dynamic scattering and electron beam channeling [38], by comparing local state descriptors obtained from a library of simulated images, for instance, as a function of thickness, to those local descriptors extracted from experimental data.

Modern imaging modalities such as STEM are hyperspectral in nature, where in addition to atomic resolution images (by Z-contrast), a full electron energy-loss spectrum can be acquired. In the case of STM, tunneling spectroscopy can be performed to measure the full electronic density of states. As such, incorporating this additional information into the feature detection/description method is an important task that should be explored in future work [39], to construct descriptors that are more physics based, thereby taking full advantage of all the information present in modern imaging modalities. This would benefit, in particular, atomic imaging modalities, such as atom probe tomography, that provide a full three-dimensional view of a material’s structure [40].

## Conclusion

In summary, we have explored a novel approach by which one can detect, identify, and classify local structural states in spatially resolved atomic images. We showed that the principles of scale invariance and contextual structural state identification, defined based on neighboring intensity distributions, give an efficient and discriminative approach by which one can extract and identify local states without the assumptions of symmetry, and illustrated the application of this method to simulated and experimental images from electron microscopy and scanning tunneling microscopy. Moreover, we showed that the more primitive concept of a structural state is sufficient to extract the salient structural configurations present in atomic imaging of materials. We foresee that our approach may provide a natural and powerful method by which one can express more complex structural correlations such as those present in frustrated and disordered systems, correlations that may lie obscured by the rigid assumptions of classical crystallography in two dimensions.

## Additional file

**Additional file 1: Figure S1.** Detected Keypoints from all atomic columns present in the simulated. STEM of a SrTiO_3_/BaTiO_3_. In the main text, only Sr, Ti, and Ba columns are included to simplify the analysis of noise dependency and classification. The addition of detected oxygen columns shown above does not modify the results in the main text. **Figure S2.** Silhouette Coefficient Analysis of the Classification is shown here for the simulated STEM image of a bulk SrTiO_3_ lattice. Top is a plot of the silhouette coefficient with different number of clusters. Bottom are the 4 structural classes (Sr, Ti, O1, O2).

## Abbreviations

STEM: scanning transmission electron microscopy
STM: scanning tunneling microscopy
SIFT: scale-invariant feature transform
LoG: Laplacian of Gaussian
STO: SrTiO_3_
LCO: LaCoO_3_
BTO: BaTiO_3_

## References

[1] Pennycook SJ, Kalinin SV (2014). Microscopy: hasten high resolution. Nature.

[2] Zhou W (2012). Direct determination of the chemical bonding of individual impurities in graphene. Phys. Rev. Lett..

[3] Krivanek OL (2010). Atom-by-atom structural and chemical analysis by annular dark-field electron microscopy. Nature.

[4] Erni R (2009). Atomic-resolution imaging with a sub-50-pm electron probe. Phys. Rev. Lett..

[5] Kim YM (2012). Probing oxygen vacancy concentration and homogeneity in solid-oxide fuel-cell cathode materials on the subunit-cell level. Nat. Mater..

[6] Catalan G (2011). Flexoelectric rotation of polarization in ferroelectric thin films. Nat. Mater..

[7] Nagao K (2015). Experimental observation of quasicrystal growth. Phys. Rev. Lett..

[8] Als-Nielsen J, McMorrow D (2011). Elements of Modern X-ray Physics.

[9] Cross JO (2015). Materials characterization and the evolution of materials. MRS. Bull..

[10] Laanait N (2014). Full-field X-ray reflection microscopy of epitaxial thin-films. J. Synchrotron Radiat.

[11] Holt M (2013). Nanoscale hard X-ray microscopy methods for materials studies. Ann. Rev. Mater. Res..

[12] Keen DA, Goodwin AL (2015). The crystallography of correlated disorder. Nature.

[13] Borisevich AY (2010). Suppression of octahedral tilts and associated changes in electronic properties at epitaxial oxide heterostructure interfaces. Phys. Rev. Lett..

[14] Gai Z (2014). Chemically induced Jahn-Teller ordering on manganite surfaces. Nat. Commun.

[15] Hytch MJ, Snoeck E, Kilaas R (1998). Quantitative measurement of displacement and strain fields from HREM micrographs. Ultramicroscopy.

[16] Belianinov A (2015). Identification of phases, symmetries and defects through local crystallography. Nat. Commun.

[17] Szeliski R (2011). Computer vision—algorithms and applications.

[18] Bishop C (2006). Pattern recognition and machine learning.

[19] Ward JH (1963). Hierarchical Grouping to Optimize an Objective Function. J. Am. Stat. Assoc.

[20] Mikolajczyk K, Schmid C (2005). A performance evaluation of local descriptors. IEEE Trans. Pattern Anal. Mach. Intell..

[21] Triggs, B. Detecting keypoints with stable position, orientation, and scale under illumination changes. In: Eighth European conference on computer vision. Prague (2004)

[22] Lindeberg T (1994). Scale-space theory: a basic tool for analysing structures at different scales. J. Appl. Stat.

[23] Burt PJ, Adelson EH (1983). The Laplacian pyramid as a compact image code. IEEE Trans. Commun..

[24] Lowe DG (2004). Distinctive image features from scale-invariant keypoints. Int. J. Comput. Vision.

[25] Obdrzˇa ´lek, S., Matas, J. Object recognition using local affine frames on maximally stable extremal regions. In: Ponce, J. (ed) Toward Category-Level Object Recognition, New York: Springer (2006)

[26] Kirkland EJ (1998). Advanced computing in electron microscopy.

[27] McLachlan G, Peel D (2000). Finite mixture models: wiley series in probability and mathematical statistics.

[28] Pennycook SJ (1992). Z-contrast transmission electron-microscopy—direct atomic imaging of materials. Ann. Rev. Mater. Sci..

[29] Rublee, E., et al. ORB: An efficient alternative to SIFT or SURF. In: Computer Vision (ICCV), 2011 IEEE international conference on. 2011

[30] Hastie T, Tobshirani R, Friedman J (2009). The elements of statistical learning: data mining, inference, and prediction. Springer series in statistics.

[31] He Q (2015). Towards 3D mapping of BO6 octahedron rotations at perovskite heterointerfaces, unit cell by unit cell. ACS. Nano.

[32] Humberto T (2012). The role of defects and doping in 2D graphene sheets and 1D nanoribbons. Rep. Prog. Phys..

[33] Fujii S (2014). Role of edge geometry and chemistry in the electronic properties of graphene nanostructures. Faraday Discuss..

[34] Ziatdinov M (2014). Direct imaging of monovacancy-hydrogen complexes in a single graphitic layer. Phys. Rev. B.

[35] Ugeda MM (2010). Missing atom as a source of carbon magnetism. Phys. Rev. Lett..

[36] Bistritzer R, MacDonald AH (2011). Moiré bands in twisted double-layer graphene. Proc. Natl. Acad. Sci..

[37] de Trambly Laissardière G, Mayou D, Magaud L (2010). Localization of Dirac Electrons in Rotated Graphene Bilayers. Nano. Lett.

[38] Loane RF, Xu P, Silcox J (1992). Incoherent imaging of zone axis crystals with ADF stem. Ultramicroscopy.

[39] Brown M, Hua G, Winder S (2011). Discriminative learning of local image descriptors. IEEE Trans. Pattern Anal. Mach. Intell..

[40] Amouyal Y, Schmitz G (2016). Atom probe tomography—a cornerstone in materials characterization. MRS. Bull..

[41] van der Walt S (2014). Scikit-image: image processing in Python. PeerJ.

[42] Bradski, G. Kaehler, A. Learning OpenCV: Computer Vision in C++ with the OpenCV Library. 2013: O’Reilly Media, Inc. 575

