# Supplementary material for: Identifying local structural states in atomic imaging by computer vision
Source: Adv Struct Chem Imaging. 2016 Nov 2;2(1):14. doi: 10.1186/s40679-016-0028-8 (PMC5093204; doi:10.1186/s40679-016-0028-8)
Supplement: Supplementary file 1 — Additional file 1: Figure S1. Detected Keypoints from all atomic columns present in the simulated. STEM of a SrTiO3/BaTiO3. In the main text, only Sr, Ti, and Ba columns are included to simplify the analysis of noise dependency and classification. The addition of detected oxygen columns shown above does not modify the results in the main text. Figure S2. Silhouette Coefficient Analysis of the Classification is shown here for the simulated STEM image of a bulk SrTiO3 lattice. Top is a plot of the silhouette coefficient with different number of clusters. Bottom are the 4 structural classes (Sr, Ti, O1, O2). [file 40679_2016_28_MOESM1_ESM.pdf]

Supplementary Information:
Identifying Local Structural States in Atomic Imaging by Computer Vision

Nouamane Laanait[1,2]*, Maxim Ziatdinov[1,2], Qian He[3], Albina Borisevich[1,3]
[1]Institute for Functional Imaging of Materials
[2]Center for Nanophase Materials Sciences
[3] Materials Sciences and Technology Division
Oak Ridge National Laboratory
Oak Ridge, TN 37831 USA
*Correspondence: laanaitn@ornl.gov

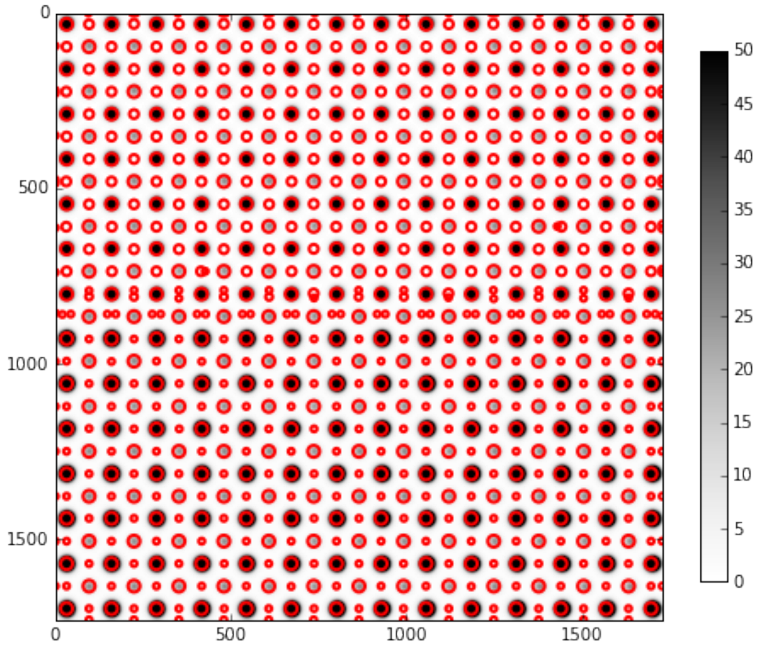

Figure S1. Detected Keypoints from all atomic columns present in the simulated STEM of a $SrTiO_3/BaTiO_3$. In the main text, only Sr, Ti, and Ba columns are included to simplify the analysis of noise dependency and classification. The addition of detected oxygen columns shown above does not modify the results in the main text.

We determined the optimal number of classes at which agglomerative clustering is truncated by calculating the silhouette coefficient [1]. The latter gives a measure of how similar a structural state S_a is to its own class $\boldsymbol{C_a}$ compared to other classes $\boldsymbol{C_i}$. The silhouette coefficient is bounded between -1 and 1, with values near -1 and 1 indicating misclassification and well-matching, respectively. For coefficient values near 0, the classification consists of many overlapping classes. To find the optimal number of well-separated structural classes, we vary the number of clusters and perform the classification, followed by computing the average silhouette coefficient for each configuration. In Fig. S2, we demonstrate this analysis for the simulated STEM image of a bulk $SrTiO_3$ lattice. The optimal number of clusters is chosen to produces the largest silhouette coefficient. Note that this analysis can only provide a statistical measure to

guide in the unsupervised classification of structural states, and is used here to demonstrate the discriminate power of the presented approach in extracting structural information from atomic imaging. In practice, incorporating a priori knowledge in the classification of structural states, through techniques of semi-supervised machine learning [2], will lead to a more effective and materials-based analysis.

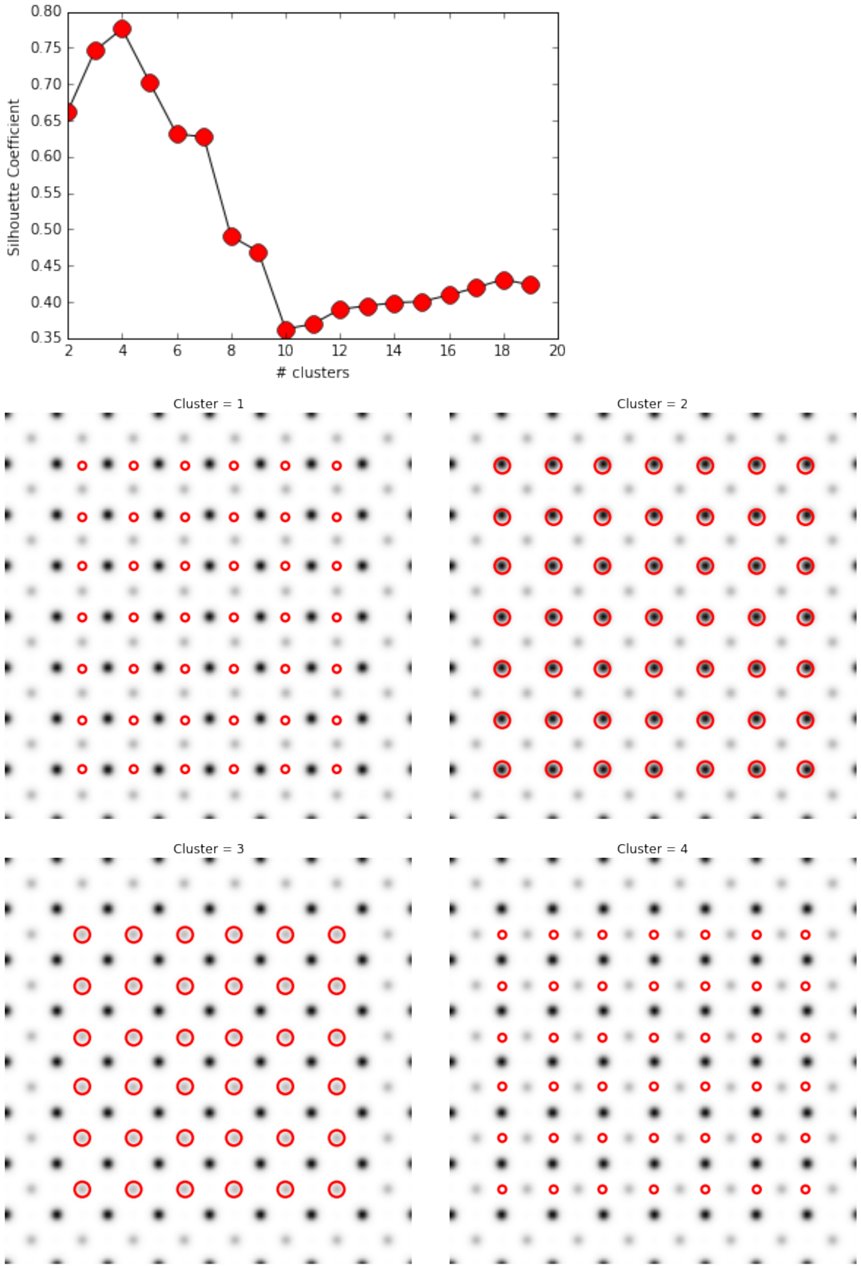

Figure S2. Silhouette Coefficient Analysis of the Classification is shown here for the simulated STEM image of a bulk $SrTiO_3$ lattice. Top is a plot of the silhouette coefficient with different number of clusters. Bottom are the 4 structural classes (Sr, Ti, O1, O2).

1. P. J. Rousseeuw, Computational and Applied Mathematics **20**, 53-65 (1987).
2. T. Hastie, R. Tobshirani and J. Friedman, *The Elements of Statistical Learning: Data Mining, Inference, and Prediction*. (Springer Science+Business Media, New York, NY, 2009).
